# Direct signaling of TL1A-DR3 on fibroblasts induces intestinal fibrosis in vivo

**DOI:** 10.1038/s41598-020-75168-5

**Published:** 2020-10-23

**Authors:** Noam Jacob, Kotaro Kumagai, Jay P. Abraham, Yosuke Shimodaira, Yuefang Ye, Justin Luu, Anna Y. Blackwood, Sofi L. Castanon, Dalton T. Stamps, Lisa S. Thomas, Rivkah Gonsky, David Q. Shih, Kathrin S. Michelsen, Stephan R. Targan

**Affiliations:** 1 Vatche and Tamar Manoukian Division of Digestive Diseases, Department of Medicine, David Geffen School of Medicine, University of California Los Angeles, 10945 Le Conte Ave., Suite 2114, Los Angeles, CA 90095 USA; 2 Division of Gastroenterology, Hepatology and Parenteral Nutrition, VA Greater Los Angeles Healthcare System, Los Angeles, CA 90073 USA; 3 F. Widjaja Foundation, Cedars-Sinai Medical Center, Inflammatory Bowel & Immunobiology Research Institute, Los Angeles, CA 90048 USA

**Keywords:** Cytokines, Inflammation, Immunology, Gastroenterology, Inflammatory bowel disease

## Abstract

Tumor necrosis factor-like cytokine 1A (TL1A, *TNFSF15*) is implicated in inflammatory bowel disease, modulating the location and severity of inflammation and fibrosis. TL1A expression is increased in inflamed mucosa and associated with fibrostenosing Crohn’s disease. Tl1a-overexpression in mice causes spontaneous ileitis, and exacerbates induced proximal colitis and fibrosis. Intestinal fibroblasts express Death-receptor 3 (DR3; the only know receptor for TL1A) and stimulation with TL1A induces activation in vitro. However, the contribution of direct TL1A-DR3 activation on fibroblasts to fibrosis in vivo remains unknown. TL1A overexpressing naïve T cells were transferred into *Rag*^*−/−*^ , *Rag*^*−/−*^ mice lacking DR3 in all cell types (*Rag*^*−/−*^*Dr3*^*−/−*^), or *Rag*^*−/−*^ mice lacking DR3 only on fibroblasts (*Rag*^*−/−*^*Dr3*^*∆Col1a2*^) to induce colitis and fibrosis, assessed by clinical disease activity index, intestinal inflammation, and collagen deposition. *Rag*^*−/−*^ mice developed overt colitis with intestinal fibrostenosis. In contrast, *Rag*^*−/−*^*Dr3*^*−/−*^ demonstrated decreased inflammation and fibrosis. Despite similar clinical disease and inflammation as *Rag*^*−/−*^*, Rag*^*−/−*^*Dr3*^*∆Col1a2*^ exhibited reduced intestinal fibrosis and attenuated fibroblast activation and migration. RNA-Sequencing of TL1A-stimulated fibroblasts identified Rho signal transduction as a major pathway activated by TL1A and inhibition of this pathway modulated TL1A-mediated fibroblast functions. Thus, direct TL1A signaling on fibroblasts promotes intestinal fibrosis in vivo. These results provide novel insight into profibrotic pathways mediated by TL1A paralleling its pro-inflammatory effects.

## Introduction

Death Domain Receptor 3 (DR3; TNFRSF25) is the only know receptor for TL1A (TNFSF15)^1–3^. This cytokine-receptor pair controls a vast array of cellular effects, including propagation of inflammatory functions, adaptive immune cell expansion, and regulation of cell development and apoptosis: DR3 can upregulate NF-KB-dependent anti-apoptotic proteins such as c-IAP2^4^. Furthermore, it can promote T cell inflammatory responses and cytokine production^5–7^. In contrast, DR3’s developmental and regulatory functions include induction of FADD- and caspase-8-dependent apoptosis and thymic negative selection^8,9^. These diverse functions are compounded by the variety of cells that can elaborate TL1A in response to multiple stimuli: dendritic cells/macrophages responding to bacterial and immune complexes; endothelial cells induced by IL-1β and TNFα; as well as lymphoid lineage cells^10–13^.

Pertaining to IBD, genetic variants of *TNFSF15* show elevated TL1A host expression, and are associated with a severe Crohn’s Disease (CD) phenotype that features intestinal fibrostenosis and need for surgery^14–16^. Consistent with human IBD, TL1A transgenic mice develop spontaneous ileitis and intestinal collagen deposition that is exaggerated under colitigenic conditions and results in severe inflammation and fibrosis^17–19^. Neutralizing anti-TL1A monoclonal antibodies attenuate these effects, reducing inflammation severity and reversing colonic fibrosis with reduced colonic fibroblast activation and lower expression of CTGF, TGFβ1 and IGF-1^20,21^.

Concerning the potential profibrotic effects of TL1A-DR3 signaling, pan-DR3-deficient mice harbor fewer colonic fibroblasts and decreased fibroblast activation, suggesting that DR3 is required for fibroblast development and function^21^. It has not been determined, however, if the reductions in fibroblast activation with pan-DR3-dificiency are due to absence of *direct* TL1A-DR3 signaling on fibroblasts, rather than due to that on other cells such as regulatory T cells, which can activate fibroblasts through the TGFβ pathway. DR3 signaling on Tregs promotes their proliferation and DR3-deficient mice have fewer Tregs^22,23^. It is therefore possible that deletion of DR3 on T cells may partly explain the reduction in myofibroblasts previously observed in *Dr3*^*−/−*^ mice^21^.

To evaluate the contribution of *direct* TL1A-DR3 signaling on fibroblasts to TL1A-mediated fibrosis in vivo, we used the adoptive T cell transfer model of colitis with TL1A over-expressing naïve T cells (Tl1a-Tg) in fibroblast-selective DR3-deficient, *Rag*^*−/−*^ mice (*Rag*^*−/−*^*Dr3*^*∆Col1a2*^). DR3-sufficient-, and pan-DR3-deficient-*Rag*^*−/−*^ mice were used as controls. In contrast to DR3-deficient hosts, which displayed attenuated clinical and histological disease parameters, *Rag*^*−/−*^*Dr3*^*∆Col1a2*^ recipients demonstrated a severe inflammatory disease phenotype. Despite this inflammation, however, selective fibroblast-loss of DR3 mitigated intestinal fibrosis and decreased activation of fibroblasts and fibroblast functions. These data demonstrate that in vivo, loss of direct TL1A-DR3 signaling on fibroblasts reduces TL1A-mediated intestinal fibrosis even in the presence of significant inflammation. These results may have important ramifications for clinical fibrosis and underscore the importance of TL1A acting in parallel as a facilitator of fibrosis and promotor of inflammation.

## Results

### Recipient pan-DR3-deficiency, but not fibroblast-selective-DR3-deficiency, reduces disease activity, colonic gross pathology, and histopathological inflammation in adoptive transfer colitis

**Figure 1:**
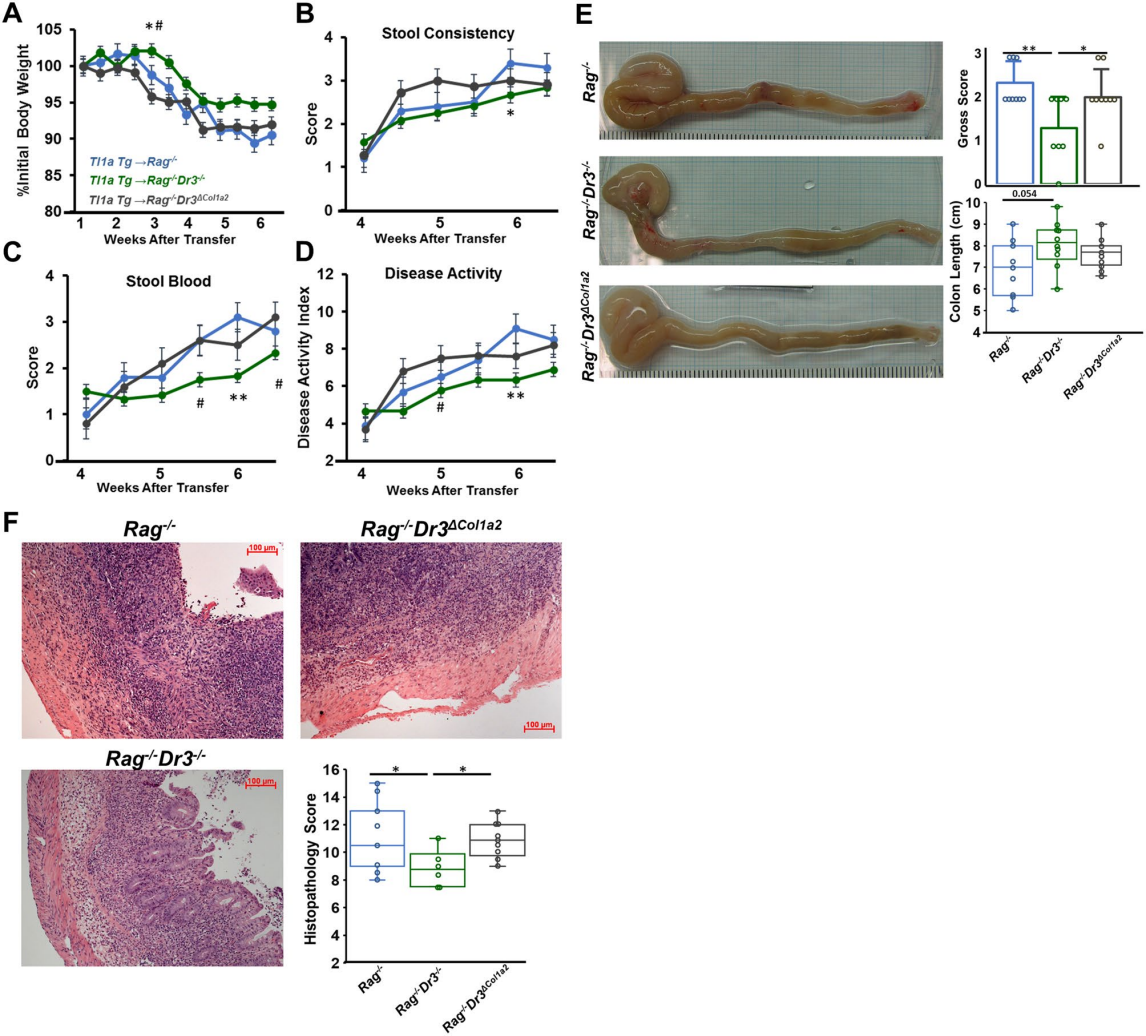
Fibroblast-selective DR3 deficiency results in severe disease activity: (**A**–**D**) Percent body weight loss, stool consistency, stool blood, and composite disease activity index are shown for the adoptive transfer model of colitis, at indicated time points for transfers of naïve Tl1a-Tg T cells into *Rag*^*−/−*^, *Rag*^*−/−*^*Dr3*^*−/−*^, or *Rag*^*−/−*^*Dr3*^*∆Col1a2*^ mice. Data are represented as means ± SEM; * indicates *p* < 0.05, **p < 0.01 *Rag*^*−/−*^*Dr3*^*−/−*^vs *Rag*^*−/−*^; # indicates *p* < 0.05 *Rag*^*−/−*^* Dr3*^*−/−*^ vs *Rag*^*−/−*^*Dr3*^*∆Col1a2*^; n = 9–10 mice per group. (**E**–**F**) Representative gross colonic specimens, quantitated macroscopic pathology scores, and colon lengths; representative H&E cecal sections and quantitated histopathological scores are shown; * indicates *p* < 0.05, **p < 0.01. Pooled data of 3 independent experiments are represented.

As TL1A overexpression promotes inflammation, both spontaneously and exaggerated under colitogenic conditions via adoptive T cell transfer, we sought to determine the effects of abrogating TL1A-DR3 signaling in adoptive transfer recipients. Consistent with previous studies, adoptive transfer of Tl1a-Tg naïve T cells into *Rag*^*−/−*^ recipients resulted in significant weight-loss beginning at week 3 after transfer. This weight-loss was accompanied by development of loose and bloody stools, and reflected in a high disease activity index (DAI) (Fig. 1A–D). Compared with *Rag*^*−/−*^ recipients, *Rag*^*−/−*^*Dr3*^*−/−*^ recipients displayed decreased severity in these disease parameters, with a reduction in initial weight-loss and significantly less loose stools, stool blood and DAI by week 6 after transfer.

The reduction in clinical disease activity imparted by pan-DR3-deficient recipients was mirrored in their gross- and histopathology scores. *Rag*^*−/−*^ recipients developed notable colonic edema, hyperemia, and shortening in the setting of TL1A-mediated colitis. This gross pathology was most pronounced in the cecum, consistent with the previously documented proximal drift imparted by TL1A-exacerbated colitis. In contrast, *Rag*^*−/−*^*Dr3*^*−/−*^ recipients had notably reduced gross pathology and a trend towards less colonic shortening (Fig. 1E).

As the cecum was most involved on gross pathology, and in prior studies, we next evaluated microscopic histopathological inflammation by H&E. *Rag*^*−/−*^ recipients developed severe inflammatory cell infiltrates extending into the submucosa and transmurally, along with widespread crypt architectural destruction. *Rag*^*−/−*^*Dr3*^*−/−*^ recipients developed a more moderate degree of cellular infiltrates and, although crypt architectural distortion was noted, it was not as extensive as in DR3-intact *Rag*^*−/−*^ recipients (Fig. 1F). These data demonstrate that in the context of TL1A-mediated exacerbated colitis, recipient DR3-deficiency mitigates clinical and histopathological disease elements.

To evaluate the direct effects of TL1A-DR3 signaling on fibroblasts in vivo in the context of experimental IBD, we generated *Rag*^*−/−*^ mice with selective DR3-deficiency on fibroblasts (*Rag*^*−/−*^*Dr3*^*∆Col1a2*^) and induced TL1A-exacerbated colitis, as above. We tested the hypothesis that in virtue of selective DR3 deficiency on fibroblasts, but otherwise intact DR3 signaling on other cell types, colitis would progress as in DR3-intact *Rag*^*−/−*^ recipients. Indeed, *Rag*^*−/−*^*Dr3*^*∆Col1a2*^ recipients of TL1A-Tg T cells developed as severe clinical disease activity as *Rag*^*−/−*^ recipients; and like *Rag*^*−/−*^ recipients, demonstrated relative heightened severity of disease compared with pan-DR3-deficient recipients (Fig. 1A–D). In contrast to recipient pan-DR3-deficiency, selective DR3-deficiency did not modulate colonic gross pathology, as *Rag*^*−/−*^*Dr3*^*∆Col1a2*^ recipients displayed marked edema and hyperemia, also most notably in the cecum (Fig. 1E). As in *Rag*^*−/−*^ recipients, the gross pathology observed in *Rag*^*−/−*^*Dr3*^*∆Col1a2*^ recipients was significantly more severe than in *Rag*^*−/−*^*Dr3*^*−/−*^ recipients, and the intensity of cecal gross pathology reflected an underlying histopathology replete with inflammatory cell infiltration and crypt destruction (Fig. 1F).

### Fibroblast-selective DR3-deficiency attenuates intestinal fibrosis in adoptive transfer colitis despite significant histopathological inflammation

**Figure 2:**
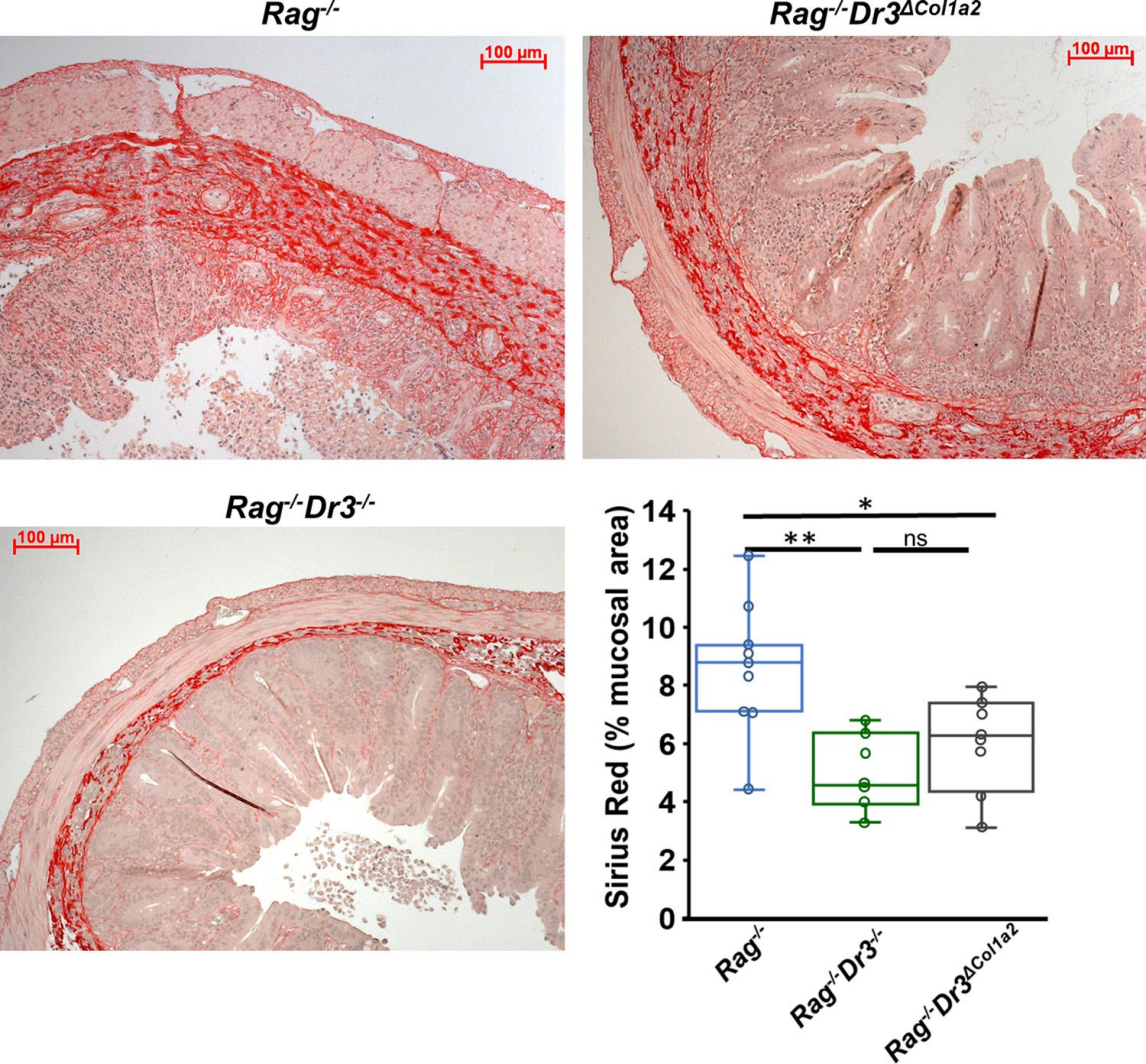
Fibroblast-selective DR3-deficiency attenuates intestinal collagen deposition: Representative Sirius red staining of collagen deposition in cecal sections and quantitated percent mucosal area by ImageJ software shown for the adoptive transfer model of colitis of *Rag*^*−/−*^, *Rag*^*−/−*^*Dr3*^*−/−*^, or *Rag*^*−/−*^*Dr3*^*∆Col1a*^ recipient mice. * indicates *p* < 0.05, **p < 0.01; ns indicates not significant. Pooled data of 3 independent experiments are represented.

While recipient pan-DR3-deficiency improved elements of disease activity and inflammation, a reduction in inflammation does not necessarily equate with a reduction in fibrosis, as has been observed in many fibrostenosing CD patients treated with steroids or immunomodulators^24,25^. However, since TL1A antagonism reduces fibrosis experimentally, we hypothesized that abrogation of TL1A-DR3 signaling in recipients would result in a reduction in collagen deposition along with the attendant improvement in inflammation noted above. Using Sirius red staining of intestinal sections, we quantified collagen deposition using Image J software (imagej.nih.gov), as previously described (and depicted in Supplementary Fig. 2)^21,36,58^. This method allows for automated color selection of Sirius Red-stained regions and provides quantitative values for the area of tissue stained. We confirmed that recipient pan-DR3-deficiency significantly reduces collagen deposition as evidenced by an average total area of 4.9% ± 1.3 Sirius Red in the cecum of *Rag*^*−/−*^*Dr3*^*−/−*^ recipients compared with 8.3% ± 2.5 in DR3-sufficient *Rag*^*−/−*^ recipients (Fig. 2). This was seen with notable reduction in thickness of submucosal collagen sections and in submucosal inflammatory cell infiltrates.

As pan-DR3-deficient recipients developed less severe inflammation and fibrosis, we asked if the effects of TL1A-mediated inflammation and fibrosis could segregate at the level of DR3 signaling on fibroblasts. We thus evaluated intestinal collagen deposition in *Rag*^*−/−*^*Dr3*^*∆Col1a2*^ recipients, which developed severe inflammatory colitis. Although there was still notable sub-mucosal area occupied by inflammatory cells, there was a decrease in the overall *density* of collagen deposition in *Rag*^*−/−*^*Dr3*^*∆Col1a2*^ recipients compared with *Rag*^*−/−*^ recipients, as shown in Fig. 2. Quantitatively, *Rag*^*−/−*^*Dr3*^*∆Col1a2*^ recipients displayed significantly less collagen deposition in the cecum, with 5.9% ± 1.7 area of Sirius red compared with 8.3% ± 2.5 in *Rag*^*−/−*^ recipients (Fig. 2). These data underscore the importance of TL1A as a fibrotic mediator acting in parallel to its pro-inflammatory role. They further suggest that TL1A may have specific effects depending on cell-type. Thus, abrogation of direct TL1A signaling on fibroblasts significantly reduces fibrosis despite pronounced inflammation resulting from TL1A overexpression and signaling on other cell-types.

### Fibroblast-selective DR3-deficiency reduces mucosal fibroblast activation

**Figure 3:**
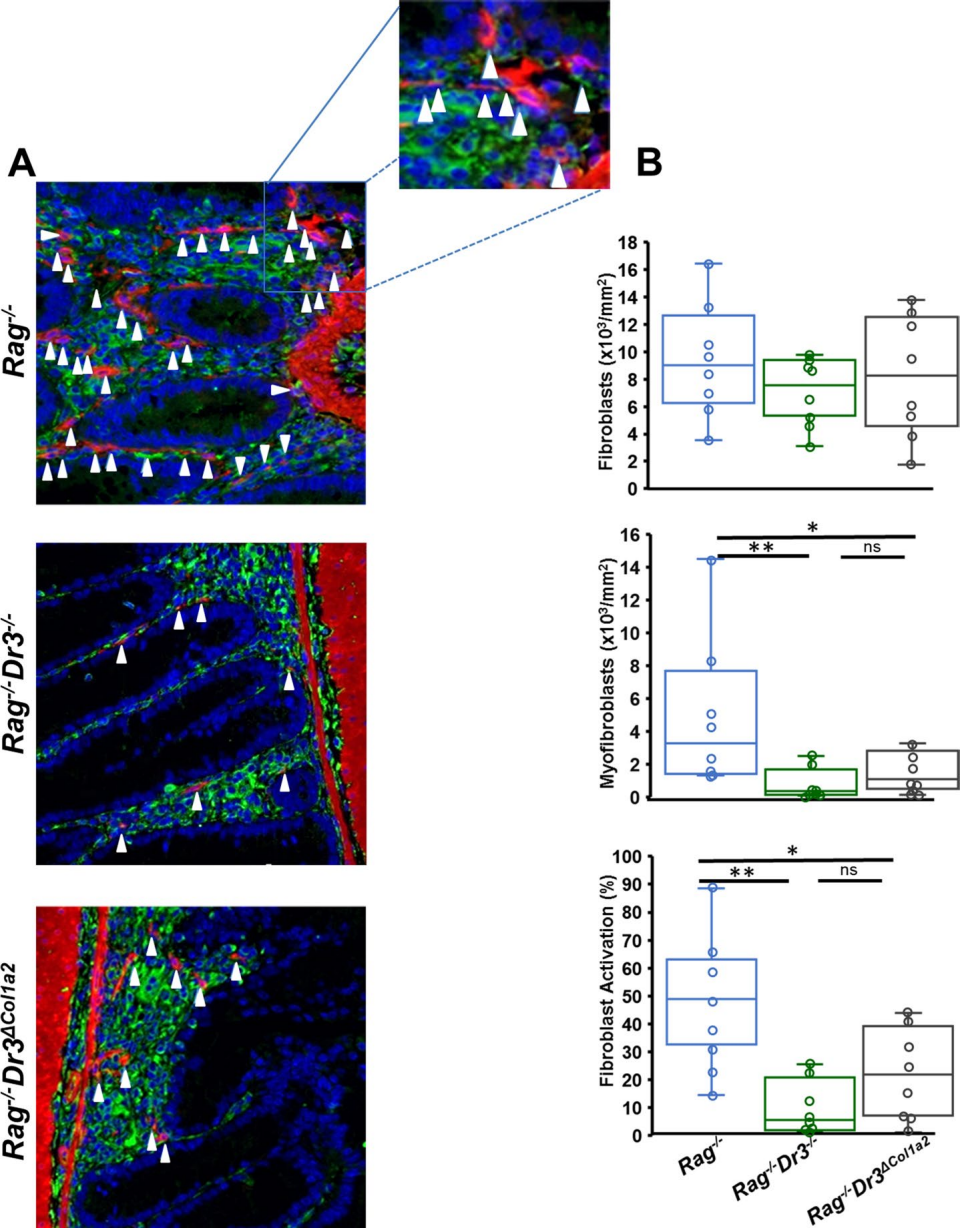
Fibroblast-selective DR3-deficiency reduces intestinal fibroblast activation: (**A**) Immunofluorescent staining of vimentin (green) and αSMA (red) from cecal sections of adoptive transfer *Rag*^*−/−*^, *Rag*^*−/−*^*Dr3*^*−/−*^, or *Rag*^*−/−*^*Dr3*^*∆Col1a2*^ recipient mice. White arrows denote myofibroblasts that co-localize vimentin and αSMA at 400× magnification. (**B**) Total numbers and percentage of myofibroblasts over total vimentin-positive cells per mm^2^ of tissue area were quantitated in an automated fashion by TissueGnostics software and displayed; * indicates *p* < 0.05, **p < 0.01; ns indicates not significant.

While TL1A induces, and pan-DR3-deficiency reduces, fibroblast activation in vitro and in vivo, the effects of TL1A-overexpression or DR3-deficiency on fibroblast activation in the context of experimental IBD has not be established. Moreover, it has not been determined if the reduction in fibroblast activation in vivo due to pan-DR3-deficiency are due to absence of direct TL1A-DR3 signaling specifically on fibroblasts, rather than due to that on other cell types that promote fibroblast activation, as mentioned earlier. To address these issues, we examined fibroblast activation in the context of fibroblast-selective DR3-deficiency under colitogenic conditions in the adoptive T cell transfer model. Consistent with previous results, pan-DR3-deficient hosts demonstrated reduced total numbers of myofibroblasts and percentage of fibroblast activation in cecal sections from colitic mice (Fig. 3). Notably, abrogation of TL1A-DR3 signaling selectively on fibroblasts also resulted in a significant reduction in myofibroblast number and relative fibroblast activation, while maintaining total fibroblast numbers. This suggests that direct TL1A-DR3 signaling on fibroblasts during colitis significantly contributes to fibroblast activation into myofibroblasts.

### Fibroblast-selective DR3-deficiency attenuates fibroblast migration ex vivo

**Figure 4:**
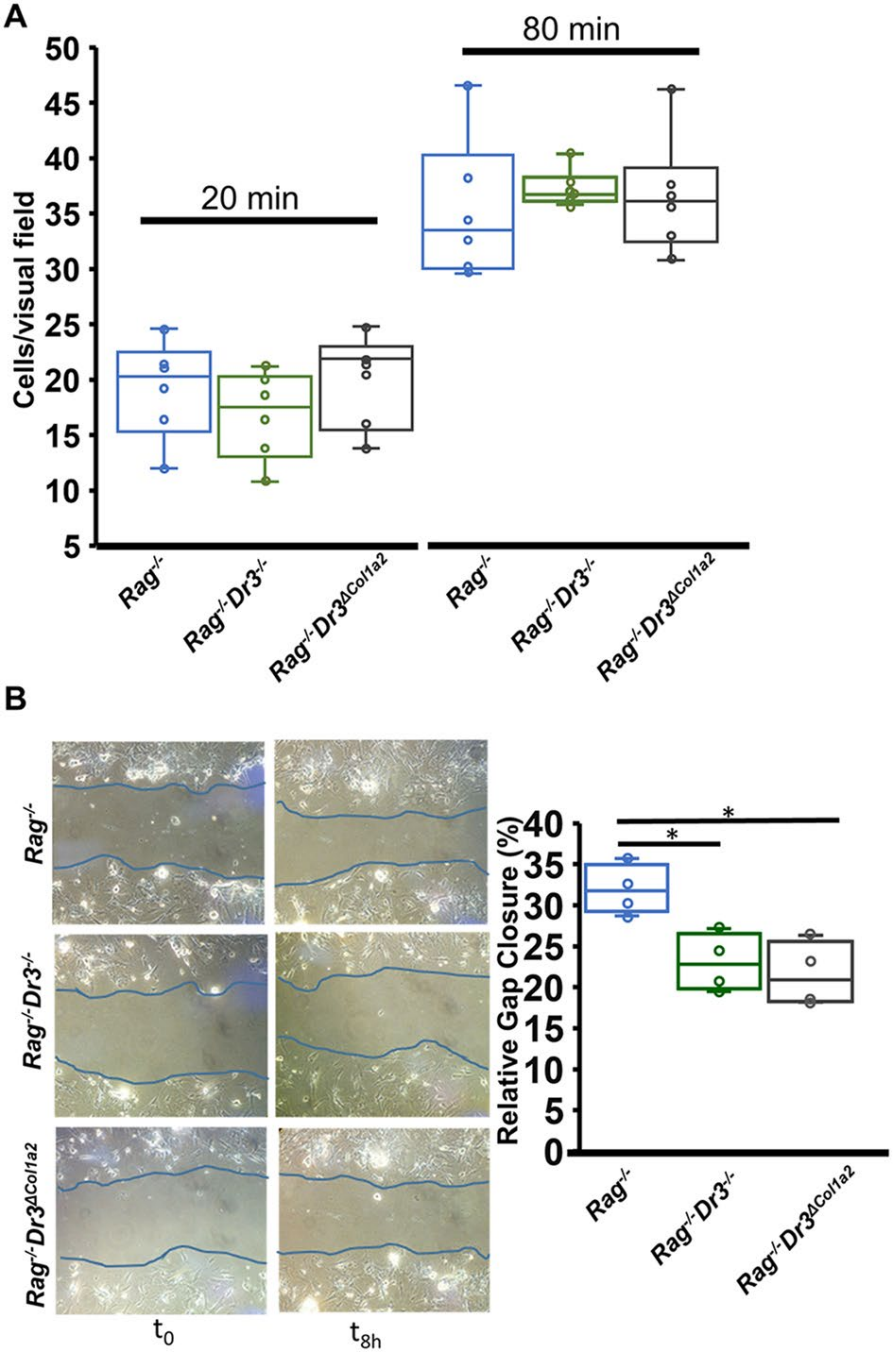
Fibroblast-selective DR3-deficiency attenuates intestinal fibroblast migration ex vivo: (**A**) Number of adherent cells/visual field indicated during adhesion assays at time points of 20 min and 80 min for fibroblasts isolated ex vivo from adoptive transfer *Rag*^*−/−*^, *Rag*^*−/−*^*Dr3*^*−/−*^, or *Rag*^*−/−*^*Dr3*^*∆Col1a2*^ recipient mice. (**B**) Representative images of gap-closure assay after simulated wound on fibroblasts isolated ex vivo from adoptive transfer *Rag*^*−/−*^, *Rag*^*−/−*^*Dr3*^*−/−*^, or *Rag*^*−/−*^*Dr3*^*∆Col1a2*^ recipient mice at initial wound (t_0_) and 8 h after migration (t_8_) with relative % area of gap closed quantitated; representative of 3 independent experiments; * indicates *p* < 0.05.

As selective DR3-deficiency reduced fibroblast activation in vivo despite the presence of significant inflammation, we sought to evaluate additional functional phenotypes of colonic fibroblasts isolated from these colitic mice ex vivo*.* The ability of effector cells to migrate to and remain in sites of disease activity has become increasingly relevant for therapeutic targeting. Adhesion and migration of fibroblasts have been used as markers of functional responses in these cells and may be relevant to their production and maintenance of fibrotic processes. We observed no differences in fibroblast adhesion after 20 min or 80 min for cells obtained from either *Rag*^*−/−*^*Dr3*^*−/−*^ or *Rag*^*−/−*^*Dr3*^*∆Col1a2*^ adoptive transfer recipients compared with *Rag*^*−/−*^ recipients (Fig. 4A).

While TL1A has been shown to enhance WT fibroblast wound closure in vitro*,* other “inflammatory” cytokines released during colitis may partly diminish fibroblast migration^26^. Thus, it was important to evaluate the migratory capacity of fibroblasts in response to TL1A-exacerbated colitis both in the context of severe inflammation seen in *Rag*^*−/−*^*Dr3*^*∆Col1a2*^ recipients, as well as during relatively reduced inflammation seen in *Rag*^*−/−*^*Dr3*^*−/−*^ recipients. Fibroblasts isolated ex vivo from colitic *Rag*^*−/−*^*Dr3*^*−/−*^ or *Rag*^*−/−*^*Dr3*^*∆Col1a2*^ recipients demonstrated significantly reduced migration 8 h after simulated wound compared with DR3-intact fibroblasts isolated from colitic *Rag*^*−/−*^ recipients (Fig. 4B). These data suggest that TL1A-DR3 activation of fibroblasts in vivo promotes functional responses during varying states of inflammation, as these differences were apparent from fibroblasts isolated ex vivo from mice with differing degrees of colitis.

### Transcriptomic analysis of Tl1a-stimulated intestinal fibroblasts identifies cytoskeletal contraction-related genes and the Rho signaling pathway

**Figure 5:**
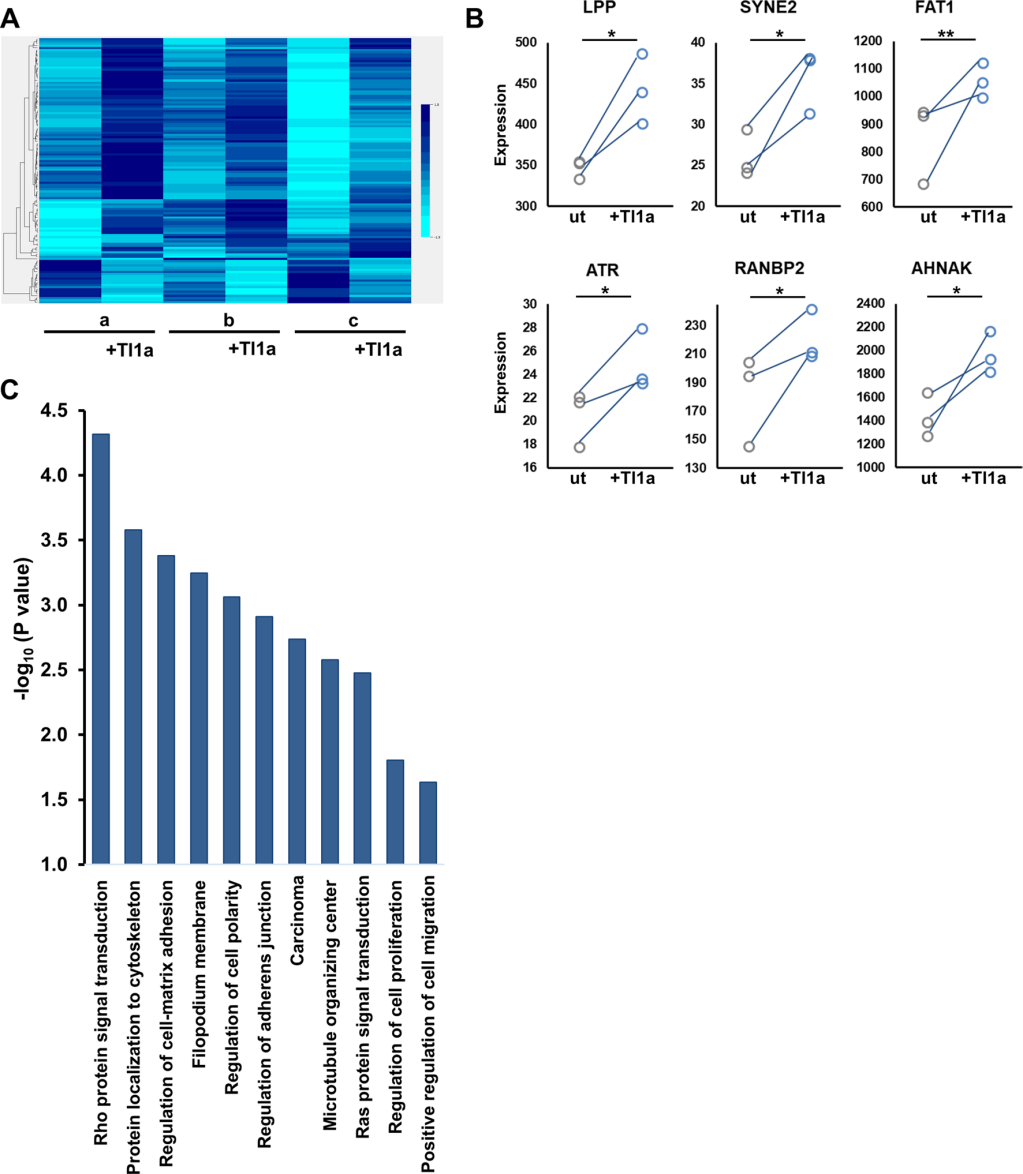
Transcriptomic analysis of Tl1a-stimulated intestinal fibroblasts identifies cytoskeletal contraction-related genes and the Rho signaling pathway: (**A**) Heatmap generated from transcriptome analysis of isolated cecal fibroblasts from three individual mice shown as untreated (a, b, c) and Tl1a-treated (a + Tl1a, etc.) pairs; BRB array tools, (brb.nci.nih.gov/BRB-ArrayTools). (**B**) Independent validation cohort was used to confirm several genes in the index group; matched pairs displayed as untreated (ut) or TL1A-treated fibroblasts with direct increase in expression for each sample shown, * indicates *p* < 0.05. (**C**) Genetic ontology pathway enrichment of differentially expressed genes induced by TL1A in (**A**).

Given that selective loss of DR3 on fibroblasts attenuated fibroblast function ex vivo in the context of TL1A overexpression, we explored potential molecular pathways by which direct TL1A signaling on fibroblasts might induce these functional effects. In previous studies, TL1A directly induced collagen expression and αSMA in cultured WT fibroblasts after short exposure of 4 h^21^. To identify pathways relevant to ex vivo assays above (and our previously published findings), we performed transcriptomic analysis of primary intestinal WT fibroblasts treated with exogenous Tl1a in vitro for 24 h. We identified a total of 168 differentially expressed genes between treated and untreated pairs; 133 up-regulated, and 35 down-regulated in response to TL1A (Fig. 5A). To validate individual genes in our initial cohort, we utilized an independent cohort of TL1A-treated and untreated pairs of primary intestinal fibroblasts isolated from age- and sex-matched wild-type mice. We confirmed significantly increased fibroblast expression of LPP, SYNE2, FAT1, AHNAK, ATR, and RANBP2 in direct response to TL1A (Fig. 5B). In previous studies, these genes have notable impact on cell migration, particularly in the context of malignancy, but they have not been demonstrated in the context of inflammatory bowel disease, intestinal fibrosis, or resulting from direct TL1A signaling^27–30^. To uncover relevant biological functions of this TL1A-induced transcriptomic profile, we performed genetic ontology enrichment analysis that yielded multiple biological processes involved in cytoskeletal organization, cell motility and contraction—most notably the Rho signal transduction pathway (Fig. 5C; Supplementary Table 1).

### Inhibition of Rho signaling pathway via Rho-associated coiled coil-forming protein kinase (ROCK) modulates intestinal fibroblast functions in vitro

**Figure 6:**
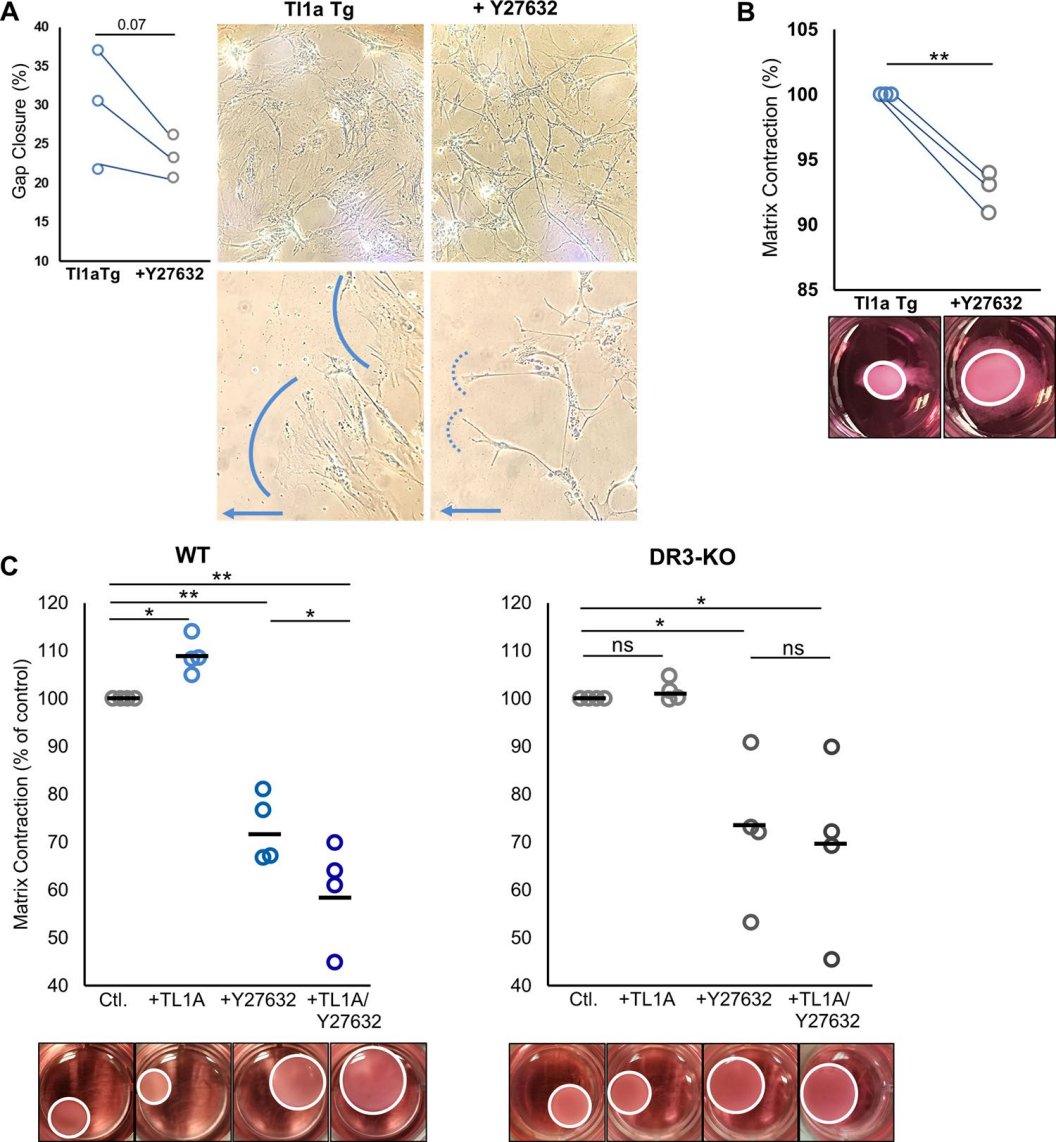
Inhibition of Rho kinase pathway modulates morphology and function of intestinal fibroblasts: (**A**) Gap closure assay and morphology of migrating intestinal fibroblasts isolated from three individual Tl1a-Tg mice, in the presence (or absence) of 25uM ROCK inhibitor Y27632, shown as treated and un-treated pairs. Blue arrow denotes direction of migration with solid vs dotted line highlighting cell extensions at migrating front; upper panels 100× objective, lower panels 200× objective. (**B**) Collagen contraction assay of intestinal fibroblasts isolated and treated as in (**A**). (**C**) Collagen contraction assay of WT or DR3^*−*/*−*^ intestinal fibroblasts treated with 100 ng/mL mouse recombinant Tl1a, 25uM ROCK inhibitor Y27632, or both. Data normalized to genotype control with bars representative of means; * indicates *p* < 0.05, **p < 0.01.

The small GTP-binding proteins belonging to the Rho family regulate signaling pathways that play key roles in formation of actin stress fibers and focal adhesions, cytoskeletal rearrangements, cell morphology, cell motility and contraction^31,32^. Rho-associated coiled coil-forming protein kinase (ROCKs) were the first downstream effectors of Rho to be discovered as important factors regulating these functions. Selective ROCK inhibition with Y-27632 impairs cell contraction, motility and cytoskeletal morphology in a variety of cell types, but has not been evaluated in the context of TL1A overexpression^33–35^. As the Rho pathway was associated in TL1A-stimulated fibroblasts, we sought to evaluate the effects of Y-27632-ROCK inhibition with regards to fibroblast functional aspects enhanced by TL1A upregulation. We initially utilized fibroblasts isolated from Tl1a-Tg mice that have been shown previously to have enhanced fibroblast functions in vitro^36^. Consistent with previously published studies, ROCK inhibition promoted a trend towards reduced migration and impacted the morphology of fibroblasts isolated from Tl1a-Tg mice, particularly noticeable in migrating cell protrusions (Fig. 6A)^33–35^. Untreated fibroblasts demonstrated wide extensions in the direction of migration with broad cellular composition, whereas when Rho kinase was inhibited, cells extended thin dendritic processes and exhibited narrow cytoskeletal networks, but still maintained interconnectivity (Fig. 6A). Considering these morphological changes and the known functions of ROCK in cytoskeletal contraction, as well as the importance of this function in fibrosis, we next evaluated the effects of ROCK inhibition in a widely-used collagen contraction assay using three-dimensional cultures of fibroblasts embedded in collagen type I gels. Gel contraction experiments revealed that ROCK inhibition significantly decreased contraction of collagen type I gel by fibroblasts isolated from Tl1a-Tg mice (Fig. 6B). To determine the direct effect of TL1A on fibroblasts in this context, WT or DR3^*−*/*−*^ fibroblasts were treated with either mouse recombinant TL1A, Y-27632-ROCK inhibitor, or both. Gel contraction experiments revealed increased collagen matrix contraction by WT fibroblasts exposed to TL1A, in contrast to DR3^*−*/*−*^ fibroblasts that showed no response to TL1A treatment (Fig. 6C). Y-27632 effectively abrogated the contraction of fibroblasts. Importantly, WT fibroblasts treated with both TL1A and Y-27632 demonstrated further reduction in contraction, suggesting that TL1A potentiates the effect of the ROCK inhibitor Y-27632 through upregulation of this pathway. Namely, as TL1A upregulates the Rho signaling pathway, there is more potential target available for Y-27632 to exert its effects. This is further affirmed by the fact that TL1A-unresponsive DR3^*−*/*−*^ fibroblasts did not show any difference in contraction when treated with Y-27632 alone vs. with Y-27632 and TL1A together (Fig. 6C). These results support the direct effect of TL1A-DR3 signaling in fibroblasts via this pathway and serve as important prospects for mechanistic links between TL1A’s direct effect on fibroblasts and intestinal fibrosis in Crohn’s disease.

## Discussion

Fibrosis often accompanies many chronic inflammatory diseases, but studies delineating the mechanisms responsible for this process and distinct pathways from inflammation are warranted. The frequency of fibrosing Crohn’s disease is significant, with approximately 40% of patients developing clinically apparent strictures throughout their lifetime^37^. Despite anti-inflammatory therapy in the form of steroids, immunomodulators, or anti-TNF agents, the frequency of fibrostenosing complications has remained significant^24,25^. Studies evaluating targetable pathways that broach *both* inflammation and fibrosis are therefore acutely relevant. We reveal a novel mechanism for TL1A-DR3 signaling directly contributing to intestinal fibrosis in parallel to its previously reported role in enhancing inflammatory responses. We provide insight into effects of TL1A on intestinal fibroblasts, demonstrating that TL1A functions directly at the level of DR3 signaling on fibroblasts by promoting activation, migration, and subsequent fibrosis (summarized in Table 1), as well as upregulating several genes involved in fibroblast functions.

**Table 1:** Summary of in vivo (adoptive transfer colitis) and ex vivo (gap closure) experiments.

Recipients	In vivo					Ex vivo
	DAI	Gross pathology	Intestinal inflammation	Intestinal fibrosis	Fibroblast activation	Fibroblast migration
*Rag*^*−/−*^	++++	++++	++++	++++	++++	++++
*Rag*^*−/−*^*Dr3*^*−/−*^	++**	++**	++*	+**	+**	++*
*Rag*^*−/−*^*Dr3*^*∆Col1a2*^	++++^#^	++++^#^	++++^#^	++*	++*	++*

+ Indicates activity of parameter measured, ranging from +: mild activity, to ++++: severe activity. *DAI* disease activity index. *Indicates *p* < 0.05, **p < 0.01 vs *Rag*^*−/−*^ ; ^#^indicates *p* < 0.05 vs *Rag*^*−/−*^*Dr3*^*−/−*^*.*

Consistent with recent reports, we have confirmed here that DR3-deficiency reduces intestinal inflammation in experimental IBD^38^. We further demonstrate that reduction in inflammation in pan-DR3 deficient recipients was accompanied by a reduction in TL1A-induced intestinal fibrosis, an important feature of experimental IBD in our model (and reflecting human CD disease risk) that had yet to be corroborated in host-DR3-deficiency. In this context, TL1A-DR3 signaling could potentially contribute to intestinal inflammation and fibrosis in both direct and indirect manners. DR3 was found to be expressed by Th2 cells and innate lymphoid cells (ILC) and was shown to enhance expansion of these cells or their ability to secrete Th2 and other pro-inflammatory cytokines. In these reports, intestinal pathology was shown to result from IL-13 produced by ILC2s upon TL1A stimulation, as well as IL-22 from ILC3s^39,40^. As the T cells transferred into pan-DR3-deficient mice were DR3-intact, this raises the possibility that part of the reduction in inflammation in *Rag*^*−/−*^*Dr3*^*−/−*^ recipients may be due to deletion of DR3 signaling on non-T cells such as ILCs, which have further been implicated recently in inflammatory models, including experimental IBD^38,41^. Moreover, the relative amount of TL1A expression in the transferred Tl1a-Tg CD4^+^ T cells has been shown to affect their pathogenicity in vivo. As these transferred T cells overexpress significant amounts of TL1A, they develop a more pronounced inflammatory, as opposed to regulatory, phenotype^42^. Adoptive transfer of DR3-deficient T cells would be useful to evaluate the contribution of DR3 signaling on T cells to intestinal inflammation and fibrosis in vivo.

While DR3 was intact on T cells in transferred mice, we also observed significant reductions in fibrosis in pan-DR3-deficient recipients, suggesting that a significant contribution to *fibrosis* in this model may be due to DR3 signaling in non-T cells. To evaluate one such potential-cell type, we generated fibroblast-selective DR3-deficient recipients. We provide novel findings that direct TL1A-DR3 signaling on fibroblasts promotes intestinal fibrosis in vivo, impacting fibroblast activation and functional phenotypes. It is notable that the effects on fibrosis contrasted inflammation, as *Rag*^*−/−*^*Dr3*^*∆Col1a2*^ recipients generated severe inflammatory responses due to TL1A overexpression, but still exhibited attenuation in intestinal collagen deposition and harbored reduced myofibroblasts that were limited in their migratory function.

The source of TL1A overexpression in our study was due to transfer of TL1A-Tg T cells. TL1A can be produced by various cell types such as macrophages, dendritic cells, and endothelial cells^3,5,43^. Interestingly, structural cells central to tissue remodeling, such as fibroblasts, may produce TL1A itself. Given their expression of DR3, TL1A production by fibroblasts could promote an autocrine feedback loop whereby they drive their own fibrotic processes independent of stimulation by adaptive immune cells. Indeed, synovial fibroblasts and intestinal myofibroblasts were found to be capable of producing TL1A after stimulation with inflammatory cytokines TNF-α or IL-1β^44,45^. Further studies with selective deletion of TL1A in fibroblasts may shed light on this potential contribution.

The most severe inflammation and fibrosis noted was in the cecum, consistent with our previous finding of fibrosis in TL1A overexpressing mice. In previous studies, the cecum was also the site of significant microbial dysbiosis where pro-fibrotic bacteria capable of affecting fibroblast phenotype were abundant^36^. As we have shown, the microbiome and TL1A both affect fibroblast functions. Studies evaluating the effect of the microbiome on fibrosis in the context of fibroblast-specific DR3-deficiency would therefore be highly informative to expand our understanding of these contributors to intestinal fibrosis.

With regards to direct cell-intrinsic effects of direct TL1A-DR3 signaling on fibroblasts, transcriptomic analysis of primary intestinal fibroblasts treated with TL1A yielded differential expression of highly relevant genes and biological processes consistent with our observed in vivo and ex vivo results. We identified several novel TL1A-induced genes/pathways in intestinal fibroblasts associated with cytoskeletal organization, contraction, and motility. Accordingly, Lipoma-preferred partner, *LPP,* was recently associated with reduced survival and increased cancer-associated fibrosis in patients with ovarian cancer as it increases focal adhesion, stress fiber formation, and motility^27,28^. Spectrin repeat containing nuclear envelope protein 2, *SYNE2*, a nuclear outer membrane protein that binds cytoplasmic F-actin, is associated with muscular dystrophies and colorectal cancer^46^. Primary fibroblasts isolated from patients affected by SYNE2 mutations exhibited alterations in migration and other physiological properties^30^. Atypical cadherin 1, *FAT1*, is a member of the cadherin superfamily and functions as an adhesion molecule. *AHNAK* (also known as desmoyokin), is a scaffold protein that affects cell contractility via its interaction with F-actin. In previous studies, knockdown of either FAT1 or AHNAK significantly reduced cell migration and invasion^29,47^. Considering the cellular functions associated with these genes, their upregulation by TL1A serves to elucidate novel pro-fibrotic pathways in intestinal fibroblasts.

Importantly, Rho kinase signal transduction was the most significantly associated biological process in our analysis, and its inhibition attenuated TL1A-enhanced fibroblast function in vitro. This pathway has been investigated in cancer biology and recently in intestinal fibrosis^48^. In experimental colitis, ROCK inhibition reduced fibrosis by inhibiting myofibroblast accumulation, expression of profibrotic factors, and accumulation of fibrotic tissue without affecting clinical disease activity or histological inflammation—a similar disjunction between inflammation and fibrosis that we observed in our study. Notably, ROCK inhibition was effective at reducing *both* inflammation and fibrosis only in conjunction with anti-TNF treatment, which was suggested as an initial clinical application in humans: the combination of a novel anti-fibrotic small molecule with a proven anti-inflammatory biologic^49^. An attractive potential alternative, however, might be monotherapeutic targeting of TL1A, as it may impact downstream pathways relevant to intestinal fibrosis such as Rho signaling, as well as previously described inflammatory pathways, thereby providing simultaneous anti-inflammatory and anti-fibrotic effects.

Thus, our studies reveal mechanisms that underlie TL1A-DR3 antagonism as a potential source for therapeutics aimed at mitigating inflammation and fibrosis in IBD, as well as potentially other inflammatory/fibrotic diseases. The heterogeneity of patients with IBD in terms of the extent and type of inflammatory responses that underlie their clinical symptoms is increasingly relevant. Recognizing differential expression of TL1A, as well as its receptor DR3, in different patient populations and various cell types/tissues in these patients will become paramount in determining who may benefit most from treatments targeting this pathway.

In summary, we reveal novel functions of TL1A in promoting intestinal inflammation and fibrosis in vivo and extend the knowledge regarding its mechanistic biology to highlight its direct action on intestinal fibroblasts. These findings further emphasize the importance of TL1A signaling pathways of fibrosis in parallel to those of inflammation, and support its importance as a candidate target for therapy in fibrotic and inflammatory diseases.

## Materials and methods

### Mice

LCK-CD2-Tl1a-GFP (*L-Tg*), *Dr3*^*−/−*^, *DR3f.*^*/f*^ were generated as previously described^11,18,21^. To generate *Dr3*^*∆Col1a2*^ mice, *DR3f.*^*/f*^ mice were bred to *Col1alpha2-Cre*^+^ mice^50^ generously provided by Dianhua Jiang, (Cedars Sinai Medical Center). Mice were then bred to *Rag1*^*−/−*^ mice (The Jackson Laboratory, Sacramento, CA) to generate *Rag*^*−/−*^*Dr3*^*−/−*^ or *Rag*^*−/−*^*Dr3*^*∆Col1a2*^ mice for adoptive transfer experiments. All mouse models were on C57BL/6 background.

### Induction of chronic colitis, disease activity indexs, macroscopic and histopathological analyses

The adoptive-transfer model of colitis was induced by intraperitoneal injection of 500,000 CD4^+^CD45RB^hi^ naive T cells isolated from L-Tg mice to *Rag1*^*−/−*^ mice; *Rag*^*−/−*^*Dr3*^*−/−*^ or *Rag*^*−/−*^*Dr3*^*∆Col1a2*51^. Mice used were age-matched, male littermates co-housed under specific pathogen-free conditions in the Animal Facility at Cedars-Sinai Medical Center (CSMC).

DAI was calculated as in previously published studies^19^. DAI score was determined twice a week for the adoptive-transfer model. Macroscopic evidence of inflammation was scored blinded to the mice genotype using the established classification^20,51^. Mucosal area of collagen deposition was identified by Sirius red staining using the NovaUltra Sirius Red Stain Kit according to manufacturer’s protocol (IHC World, Woodstock, MD). Stained gut sections were quantitated for the relative degree of fibrosis using ImageJ software (imagej.nih.gov), as previously described^21^.

### Fibroblast assays

Mouse primary colonic fibroblasts were isolated, and gap closure and adhesion assays performed, as previously described^21,36^: Equal numbers of fibroblasts per group (1 × 10^5^ cells) were seeded in 8 chamber slides and cultured for 24–48 h until a monolayer was formed. A scratch was created with a P200 pipette tip. Cell debris was removed by washing cells with PBS and then cell-culture medium was replaced with time-lapse images taken after 8 h under an Olympus CK2 microscope at 100× objective. The area of the gap between the two migrating fronts of the cells was quantified using ImageJ software v1.53a and relative percent area of gap closed at the indicated time points was calculated as (area t_0_ − area t_x_)/area t_0_. For inhibitor assays, ROCK inhibitor Y27632 (Stemcell Technologies, Cambridge, MA) was added at a concentration 25 μM, as in recent publications^34^, for 12 h prior to simulated wound and then maintained during the migration period.

For fibroblast adhesion assays, an equal number of cells were seeded into 24-well plates and allowed to settle for either 20 or 80 min, after which the wells were washed twice with PBS to remove non-adherent cells. Adherent cells were counted for 5 visual fields/well (representing four quadrants and the center of the well) at 200× magnification, then averaged. The average number of adherent cells per visual field is then displayed for each well, as described previously^36^.

Collagen gel contraction assays (Cell Biolabs, Inc, San Diego, CA) were performed according to the manufacturer’s protocol: Collagen cell contraction matrix was prepared by adding 2 parts cell suspension (at a concentration of 2 × 10^6^ cells/mL of primary intestinal fibroblasts) and 8 parts of cold collagen gel working solution. 0.5 mL of the cell contraction matrix was added to each well of a 24-well Cell Contraction Plate. The plate was then incubated at 37 °C and 5% CO_2_ for 1 h. After collagen polymerization, 1.0 mL of culture medium, with/without mouse recombinant Tl1a (R&D Systems, Minneapolis, MN) at a concentration 100 ng/mL, or ROCK inhibitor Y27632 (Stemcell Technologies, Cambridge, MA) at a concentration 25 μM^34^, was added to the top of each collagen gel lattice. Contraction was monitored for 16–24 h and the collagen gel size change (contraction index) was imaged and calculated with ImageJ software.

### Histological myofibroblast quantification

Fibroblast and myofibroblasts were quantified by anti-vimentin and anti-α-Smooth Muscle Actin immunofluorescence-stained OCT tissue sections. 4 µm frozen sections were fixed with 10% formalin, blocked in 10% BSA, 0.1% Triton X-100 TBST, and stained overnight at 4 °C with primary antibodies: rabbit polyclonal anti-αSMA Ab (Abcam, Cambridge, MA) at 1:100 dilution and chicken polyclonal anti-Vimentin Ab (Abcam, Cambridge, MA) at 1:2000 dilution. Secondary antibody at 1:500 dilution was added for 2 h at room temperature with donkey anti-rabbit IgG-Alexa-fluor-647 and goat anti-chicken IgY-DyLight 488 (Abcam, Cambridge, MA). Fluorescent images were captured by the CSMC Imaging Core and analyzed using TissueGnostics TissueFAXS 200 system (Tissue Gnostics GmbH, Vienna, Austria) for cell-based counting of automatically recognized positive cells in a FACS-like manner of scattergram analysis, as described previously^52,53^.

### Transcriptome profiling of Tl1a-stimulated fibroblasts and statistical analysis

Mouse primary intestinal fibroblasts were isolated, as above, and cultured with or without mouse recombinant Tl1a at a concentration 100 ng/mL for 24 h. Total RNA was isolated from cultured fibroblasts using Qiagen RNeasy Micro Kit according to the manufacturer’s protocol. RNAseq was performed by the UCLA Technology Center for Genomics & Bioinformatics (TCGB, Los Angeles, CA). Libraries for RNA-Seq were prepared with Kapa Stranded Kit. The workflow consisted of mRNA capture, cDNA generation, and end repair to generate blunt ends, A-tailing, adaptor ligation and PCR amplification. Different adaptors were used for multiplexing samples in one lane. The data was sequenced on Illumina NextSeq500 for a single-read 50 bp run. Data quality check was done on Illumina SAV. Demultiplexing was performed with Illumina Bcl2fastq2 v 2.17 program. The reads were mapped to the latest UCSC transcript set using Bowtie2 version 2.1.0^54^ and the gene expression level was estimated using RSEM v1.2.15^55^. TMM (trimmed mean of M-values) was used to normalize the gene expression. Gene expression data were processed using BRB array tools (brb.nci.nih.gov/BRB-ArrayTools) and lumi package in R. The data were log2-transformed and normalized using quantile normalization. Genes with data missing exceeding 50% or a log-ratio variation less than 80 percent were filtered out. Differential gene expression was determined by univariate paired T-test (with random variance model parameters: a = 1.65119, b = 38.60092, Kolmogorov–Smirnov statistic = 0.01463) and percent of exact multivariate permutations test was computed based on 1000 available permutations and a maximum allowed local false discovery rate < 0.2. Erichr: https://amp.pharm.mssm.edu/Enrichr/ was used for pathway enrichment analysis of differentially expressed genes^56,57^. An independent validation cohort of matched pairs of fibroblasts was treated and sequenced as above, and used to confirm several genes in the index group. Tests for statistical significance were determined using JMP Statistical Software (Cary, NC). Matched pair comparisons (Student’s paired T test) for normalized distributions of TMM values was performed, with Robust-fit regression analysis used in the case of non-normalized distributions. Clinical and histological group differences tested using standard methods depending on variables measured: Student’s t-test for comparisons between two groups or matched pairs, Mann Whitney test for comparisons between two groups requiring non-parametric testing. When indicated, 1-way Analysis of Variance (ANOVA) with Tukey’s honestly significant difference (HSD) test for multiple comparisons was used. In all settings, a *p* value of less than 0.05 indicated a statistically significant difference in the parameter being compared.

### Study approval

This study was carried out in strict accordance with the Guide for the Care and Use of Laboratory Animals of the National Institutes of Health. Animal studies were approved by the CSMC Animal Care and Use Committee, under IACUC protocol 4942.

## Supplementary information

Supplementary Information

## References

[1] Chinnaiyan AM (1996). Signal transduction by DR3, a death domain-containing receptor related to TNFR-1 and CD95. Science.

[2] Kitson J (1996). A death-domain-containing receptor that mediates apoptosis. Nature.

[3] Migone TS (2002). TL1A is a TNF-like ligand for DR3 and TR6/DcR3 and functions as a T cell costimulator. Immunity.

[4] Wen L (2003). TL1A-induced NF-kappaB activation and c-IAP2 production prevent DR3-mediated apoptosis in TF-1 cells. J. Biol. Chem..

[5] Castellanos JG (2018). Microbiota-induced TNF-like ligand 1A drives group 3 innate lymphoid cell-mediated barrier protection and intestinal T cell activation during colitis. Immunity.

[6] Meylan F (2008). The TNF-family receptor DR3 is essential for diverse T cell-mediated inflammatory diseases. Immunity.

[7] Pappu BP (2008). TL1A-DR3 interaction regulates Th17 cell function and Th17-mediated autoimmune disease. J. Exp. Med..

[8] Varfolomeev EE (1998). Targeted disruption of the mouse Caspase 8 gene ablates cell death induction by the TNF receptors, Fas/Apo1, and DR3 and is lethal prenatally. Immunity.

[9] Wang EC (2001). DR3 regulates negative selection during thymocyte development. Mol. Cell. Biol..

[10] Al-Lamki RS (2008). TL1A both promotes and protects from renal inflammation and injury. J. Am. Soc. Nephrol..

[11] Bamias G (2006). Role of TL1A and its receptor DR3 in two models of chronic murine ileitis. Proc. Natl. Acad. Sci. U.S.A..

[12] Prehn JL (2007). The T cell costimulator TL1A is induced by FcgammaR signaling in human monocytes and dendritic cells. J. Immunol..

[13] Shih DQ (2009). Microbial induction of inflammatory bowel disease associated gene TL1A (TNFSF15) in antigen presenting cells. Eur. J. Immunol..

[14] Hirano A (2013). Association study of 71 European Crohn's disease susceptibility loci in a Japanese population. Inflamm. Bowel. Dis..

[15] Michelsen KS (2009). IBD-associated TL1A gene (TNFSF15) haplotypes determine increased expression of TL1A protein. PLoS ONE.

[16] Picornell Y (2007). TNFSF15 is an ethnic-specific IBD gene. Inflamm. Bowel. Dis..

[17] Meylan F (2011). The TNF-family cytokine TL1A drives IL-13-dependent small intestinal inflammation. Mucosal Immunol..

[18] Shih DQ (2011). Constitutive TL1A (TNFSF15) expression on lymphoid or myeloid cells leads to mild intestinal inflammation and fibrosis. PLoS ONE.

[19] Barrett R (2012). Constitutive TL1A expression under colitogenic conditions modulates the severity and location of gut mucosal inflammation and induces fibrostenosis. Am. J. Pathol..

[20] Takedatsu H (2008). TL1A (TNFSF15) regulates the development of chronic colitis by modulating both T-helper 1 and T-helper 17 activation. Gastroenterology.

[21] Shih DQ (2014). Inhibition of a novel fibrogenic factor Tl1a reverses established colonic fibrosis. Mucosal Immunol..

[22] Jia LG (2016). A novel role for TL1A/DR3 in protection against intestinal injury and infection. J. Immunol..

[23] Schreiber TH (2010). Therapeutic Treg expansion in mice by TNFRSF25 prevents allergic lung inflammation. J. Clin. Invest..

[24] Latella G, Papi C (2012). Crucial steps in the natural history of inflammatory bowel disease. World J. Gastroenterol..

[25] Bouhnik Y (2018). Efficacy of adalimumab in patients with Crohn's disease and symptomatic small bowel stricture: a multicentre, prospective, observational cohort (CREOLE) study. Gut.

[26] Drygiannakis I (2013). Proinflammatory cytokines induce crosstalk between colonic epithelial cells and subepithelial myofibroblasts: implication in intestinal fibrosis. J. Crohns Colitis.

[27] Leung CS (2018). Cancer-associated fibroblasts regulate endothelial adhesion protein LPP to promote ovarian cancer chemoresistance. J. Clin. Invest..

[28] Ngan E (2017). LPP is a Src substrate required for invadopodia formation and efficient breast cancer lung metastasis. Nat. Commun..

[29] Sohn M (2018). Ahnak promotes tumor metastasis through transforming growth factor-beta-mediated epithelial-mesenchymal transition. Sci. Rep..

[30] Taranum S (2012). LINC complex alterations in DMD and EDMD/CMT fibroblasts. Eur. J. Cell Biol..

[31] Heasman SJ, Ridley AJ (2008). Mammalian Rho GTPases: new insights into their functions from in vivo studies. Nat. Rev. Mol. Cell Biol..

[32] Ridley AJ, Hall A (1992). The small GTP-binding protein rho regulates the assembly of focal adhesions and actin stress fibers in response to growth factors. Cell.

[33] Miron-Mendoza M, Graham E, Kivanany P, Quiring J, Petroll WM (2015). The role of thrombin and cell contractility in regulating clustering and collective migration of corneal fibroblasts in different ECM environments. Invest. Ophthalmol. Vis. Sci..

[34] Wang WY, Davidson CD, Lin D, Baker BM (2019). Actomyosin contractility-dependent matrix stretch and recoil induces rapid cell migration. Nat. Commun..

[35] Zhou C, Petroll WM (2010). Rho kinase regulation of fibroblast migratory mechanics in fibrillar collagen matrices. Cell. Mol. Bioeng..

[36] Jacob N (2018). Inflammation-independent TL1A-mediated intestinal fibrosis is dependent on the gut microbiome. Mucosal Immunol..

[37] Cosnes J, Gower-Rousseau C, Seksik P, Cortot A (2011). Epidemiology and natural history of inflammatory bowel diseases. Gastroenterology.

[38] Li Z (2018). Death Receptor 3 Signaling Controls The Balance Between Regulatory And Effector Lymphocytes in SAMP1/YitFc mice with Crohn's disease-like ileitis. Front. Immunol..

[39] Longman RS (2014). CX(3)CR1(+) mononuclear phagocytes support colitis-associated innate lymphoid cell production of IL-22. J. Exp. Med..

[40] Yu X (2014). TNF superfamily member TL1A elicits type 2 innate lymphoid cells at mucosal barriers. Mucosal Immunol..

[41] Malhotra N (2018). RORalpha-expressing T regulatory cells restrain allergic skin inflammation. Sci. Immunol..

[42] Sidhu-Varma M, Shih DQ, Targan SR (2016). Differential levels of Tl1a affect the expansion and function of regulatory T cells in modulating murine colitis. Inflamm. Bowel Dis..

[43] Bamias G (2003). Expression, localization, and functional activity of TL1A, a novel Th1-polarizing cytokine in inflammatory bowel disease. J. Immunol..

[44] Bamias G (2017). Crohn's disease-associated mucosal factors regulate the expression of TNF-like cytokine 1A and its receptors in primary subepithelial intestinal myofibroblasts and intestinal epithelial cells. Transl. Res..

[45] Zhang J (2009). Role of TL1A in the pathogenesis of rheumatoid arthritis. J. Immunol..

[46] Luxton GW, Gomes ER, Folker ES, Vintinner E, Gundersen GG (2010). Linear arrays of nuclear envelope proteins harness retrograde actin flow for nuclear movement. Science.

[47] Nishikawa Y (2011). Human FAT1 cadherin controls cell migration and invasion of oral squamous cell carcinoma through the localization of beta-catenin. Oncol. Rep..

[48] Holvoet T (2017). Treatment of intestinal fibrosis in experimental inflammatory bowel disease by the pleiotropic actions of a local rho kinase inhibitor. Gastroenterology.

[49] Rieder F (2017). ROCKing the field of intestinal fibrosis or between a ROCK and a hard place?. Gastroenterology.

[50] Florin L (2004). Cre recombinase-mediated gene targeting of mesenchymal cells. Genesis.

[51] Ostanin DV (2006). T cell-induced inflammation of the small and large intestine in immunodeficient mice. Am. J. Physiol. Gastrointest. Liver Physiol..

[52] Kuo TC (2013). Angiopoietin-like protein 1 suppresses SLUG to inhibit cancer cell motility. J. Clin. Invest..

[53] Liesz A (2009). Regulatory T cells are key cerebroprotective immunomodulators in acute experimental stroke. Nat. Med..

[54] Langmead B, Salzberg SL (2012). Fast gapped-read alignment with Bowtie 2. Nat. Methods.

[55] Li B, Dewey CN (2011). RSEM: accurate transcript quantification from RNA-Seq data with or without a reference genome. BMC Bioinform..

[56] Chen EY (2013). Enrichr: interactive and collaborative HTML5 gene list enrichment analysis tool. BMC Bioinform..

[57] Kuleshov MV (2016). Enrichr: a comprehensive gene set enrichment analysis web server 2016 update. Nucleic Acids Res..

[58] Schneider CA, Rasband WS, Eliceiri KW (2012). NIH Image to ImageJ: 25 years of image analysis. Nat. Methods.

