# Supplementary material for: Direct signaling of TL1A-DR3 on fibroblasts induces intestinal fibrosis in vivo
Source: Sci Rep. 2020 Oct 23;10:18189. doi: 10.1038/s41598-020-75168-5 (PMC7584589; doi:10.1038/s41598-020-75168-5)
Supplement: Supplementary file 1 — Supplementary Information [file 41598_2020_75168_MOESM1_ESM.pdf]

Title: Direct Signaling of TL1A-DR3 on Fibroblasts Induces Intestinal Fibrosis *In Vivo*

Supplementary Information

Authors: Noam Jacob*[,2,3], Kotaro Kumagai[1], Jay P. Abraham[1], Yosuke Shimodaira[1], Yuefang Ye[1], Justin Luu[1], Anna Y. Blackwood[1], Sofi L. Castanon[1], Dalton T. Stamps[1], Lisa S. Thomas[1], Rivkah Gonsky[1], David Q. Shih[1], Kathrin S. Michelsen[1], Stephan R. Targan[1]

1. F. Widjaja Foundation, Inflammatory Bowel & Immunobiology Research Institute, Cedars-Sinai Medical Center, Los Angeles, CA 90048, USA.

2. Vatche and Tamar Manoukian Division of Digestive Diseases, Department of Medicine, David Geffen School of Medicine, University of California Los Angeles, Los Angeles, CA 90095, USA.

3. Division of Gastroenterology, Hepatology and Parenteral Nutrition, VA Greater Los Angeles Healthcare System Los Angeles, CA 90073, USA.

***Correspondence:**

Noam Jacob, MD, PhD

Division of Digestive Diseases

Department of Medicine, UCLA

10945 Le Conte Ave., Suite 2114

Los Angeles, CA 90095

Email: njacob@mednet.ucla.edu

Disclosures: The authors have declared that no conflicts of interests exist

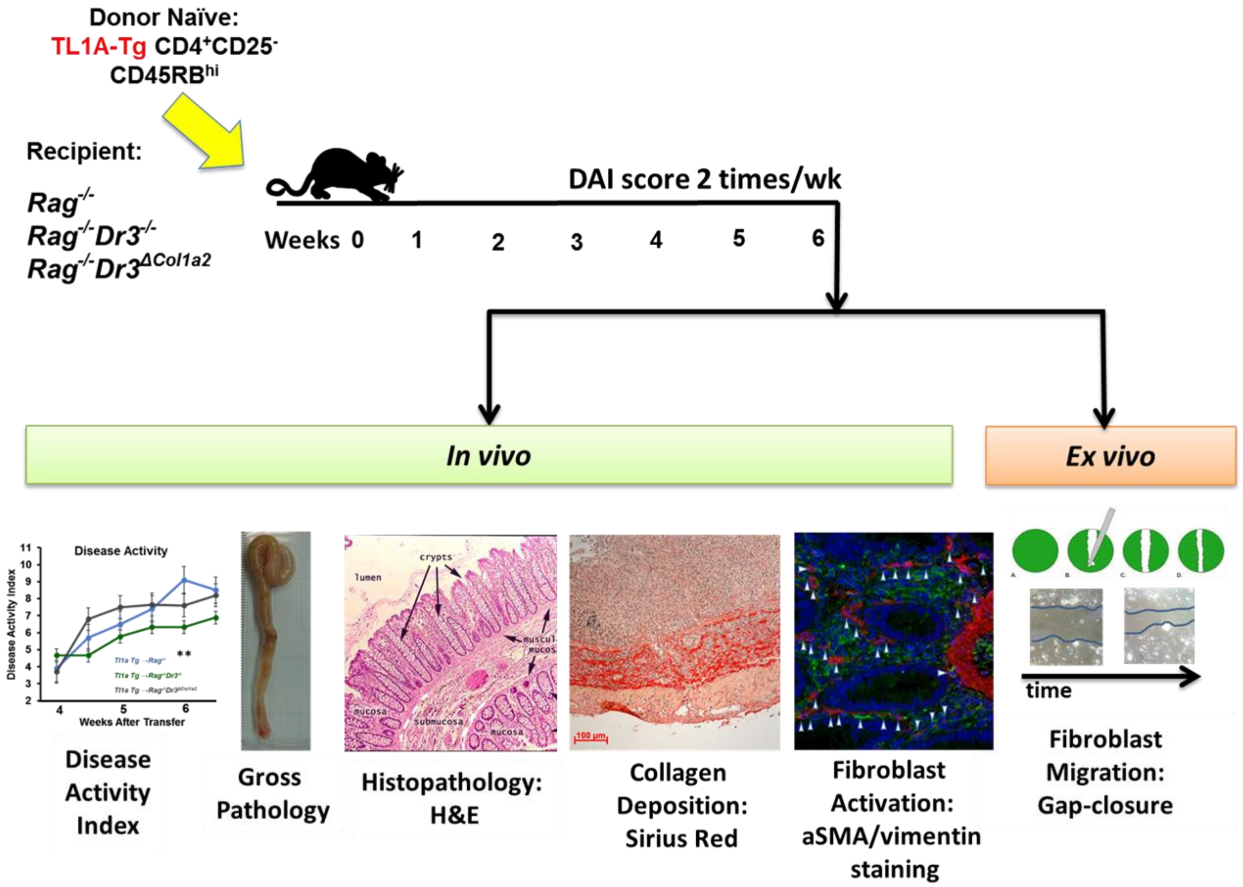

Supplementary Figure 1. *Schematic Diagram of Experimental Set-up:* Using the adoptive T cell transfer model, transfers of TL1A over-expressing naïve T cells (*Tl1a-Tg*) were used to induce intestinal inflammation and fibrosis in *Rag*$^{-/-}$ mice; *Rag*$^{-/-}$ mice lacking DR3 in all cell types (*Rag*$^{-/-}$*Dr3*$^{-/-}$); or *Rag*$^{-/-}$ mice lacking DR3 only on fibroblasts (*Rag*$^{-/-}$*Dr3*$^{\Delta Col1a2}$) (n=6-9 mice/group). Disease activity index (DAI: weight loss, stool consistency, stool blood), gross pathology of the colon, intestinal inflammation (H&E), and degree of collagen deposition (Sirius red) were assessed. Fibroblast quantification and activation in colonic sections were calculated by immunofluorescent staining with anti-Vimentin and anti-alpha-Smooth-Muscle-Actin (αSMA). Fibroblasts were isolated from the colons of colitic mice and fibroblast migration was determined by rate of gap closure in response to simulated wounds.

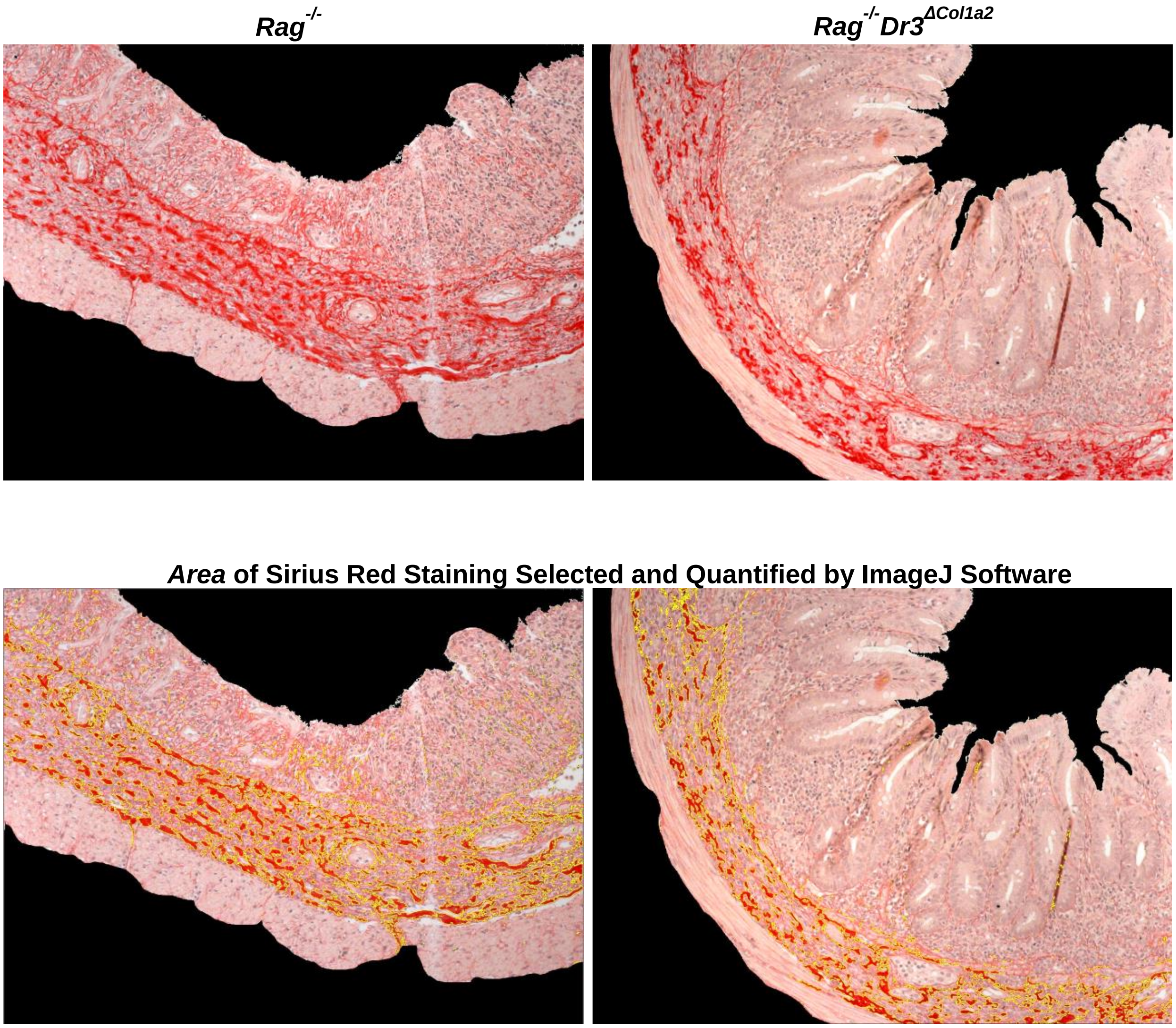

Supplementary Figure 2. *Quantification of Sirius Red:* Upper panels depict representative Sirius red staining of collagen deposition in cecal sections after being imported into ImageJ software. The area of Sirius red staining is then selected (surrounded in yellow), and quantified by ImageJ software, shown in lower panels (ImageJ v1.53a; imagej.nih.gov).

**Supplementary Table 1.** *Biological processes and associated genes upregulated by TL1A in primary intestinal fibroblasts:*	
**Biological Process**	**Genes**
Rho protein signal transduction	RHOT1; ROCK1; ARHGAP5; CTNNAL1, ARHGEF6
Protein localization to microtubule cytoskeleton	CRIPT; KIF20B
Regulation of cell-matrix adhesion	ROCK1; UTRN; GPM6B
Filopodium membrane	SYNE2; UTRN
Regulation of establishment of cell polarity	ROCK1; KIF20B
Regulation of adherens junction/actin filament-based processes	ROCK1; GPM6B
Carcinoma	LPP; SYNE2; RANBP2; AHNAK; FAT1; ATR; RNASEL; ROCK1; YTHDC2; CASC5; NCAPG; ARHGAP5; SKA3; TATDN1; XPO1; RHOT1; PDCD10; STAB1; SVEP1; CTNNAL1; CC2D2A; PITX1; CNTLN; HERC6; OFD1; VDR; STYK1; VPS13A; HAUS3; PARP14; MPEG1; ACVR2A; ASPM; KAT2B; VANGL2; MMP16; NOX4; CCDC66; UTRN; KIF20B; CDH19; ARHGEF6
Microtubule organizing center	RANBP2; OFD1; KAT2B; HAUS3; KIF20B; CNTLN
Ras protein signal transduction	RHOT1; ROCK1; ARHGAP5; CTNNAL1
Positive regulation of cell migration	SYNE2; PDCD10; KIF20B
Regulation of cell proliferation	KAT2B; NOX4; PDCD10; VDR; STYK1; KIF20B
